# Mapping coral calcification strategies from in situ boron isotope and trace element measurements of the tropical coral *Siderastrea siderea*

**DOI:** 10.1038/s41598-020-78778-1

**Published:** 2021-01-12

**Authors:** T. B. Chalk, C. D. Standish, C. D’Angelo, K. D. Castillo, J. A. Milton, G. L. Foster

**Affiliations:** 1 School of Ocean and Earth Science, University of Southampton, National Oceanography Centre Southampton, Southampton, UK; 2 Marine Sciences, University of North Carolina at Chapel Hill, Chapel Hill, NC USA

**Keywords:** Environmental sciences, Ocean sciences, Biogeochemistry, Climate sciences, Biogeochemistry

## Abstract

Boron isotopic and elemental analysis of coral aragonite can give important insights into the calcification strategies employed in coral skeletal construction. Traditional methods of analysis have limited spatial (and thus temporal) resolution, hindering attempts to unravel skeletal heterogeneity. Laser ablation mass spectrometry allows a much more refined view, and here we employ these techniques to explore boron isotope and co-varying elemental ratios in the tropical coral *Siderastrea siderea*. We generate two-dimensional maps of the carbonate parameters within the calcification medium that deposited the skeleton, which reveal large heterogeneities in carbonate chemistry across the macro-structure of a coral polyp. These differences have the potential to bias proxy interpretations, and indicate that different processes facilitated precipitation of different parts of the coral skeleton: the low-density columella being precipitated from a fluid with a carbonate composition closer to seawater, compared to the high-density inter-polyp walls where aragonite saturation was ~ 5 times that of external seawater. Therefore, the skeleton does not precipitate from a spatially homogeneous fluid and its different parts may thus have varying sensitivity to environmental stress. This offers new insights into the mechanisms behind the response of the *S. siderea *skeletal phenotype to ocean acidification.

## Introduction

Tropical coral reefs are some of the most important ecosystems on the planet, contributing greater than US$30 billion in ecosystem services each year worldwide^1^. They are diversity hotspots, offer coastal protection, and sustain important economic activities such as fisheries and tourism. All these ecosystem functions depend on the framework of the reef that is constructed in the extracellular calcifying medium (ECM)—a (sub-)micron-sized space sandwiched between each coral animal and its existing skeleton^2^. Tropical coral reefs and the ecosystems and industries they support face a number of threats from human activity, including rising ocean temperatures, ocean acidification, eutrophication, overfishing, sea level rise, and pollution. These anthropogenic stressors affect key physiological processes in reef-building Scleractinian (stony) corals, such as coral biomineralisation, which in turn impacts aspects of their skeleton (such as rate of extension, density, porosity and strength) leading to decreased fitness^3^, increased coral mortality and ultimately loss of ecosystem functionality.

Calcification within the ECM occurs from semi-isolated pools of modified seawater and the coral animal has tight control on both the carbonate system and crystal growth in this space through the deployment of enzymatic pumps (e.g. Ca-ATPase) and secretion of acid-rich proteins^4^. Tracking the state of the carbonate system in the ECM is emerging as a central methodology underpinning a mechanistic understanding of how environmental change, such as ocean acidification, influences skeletal formation and ultimately the growth of these important ecosystem engineers^5–7^.

**Figure 1:**
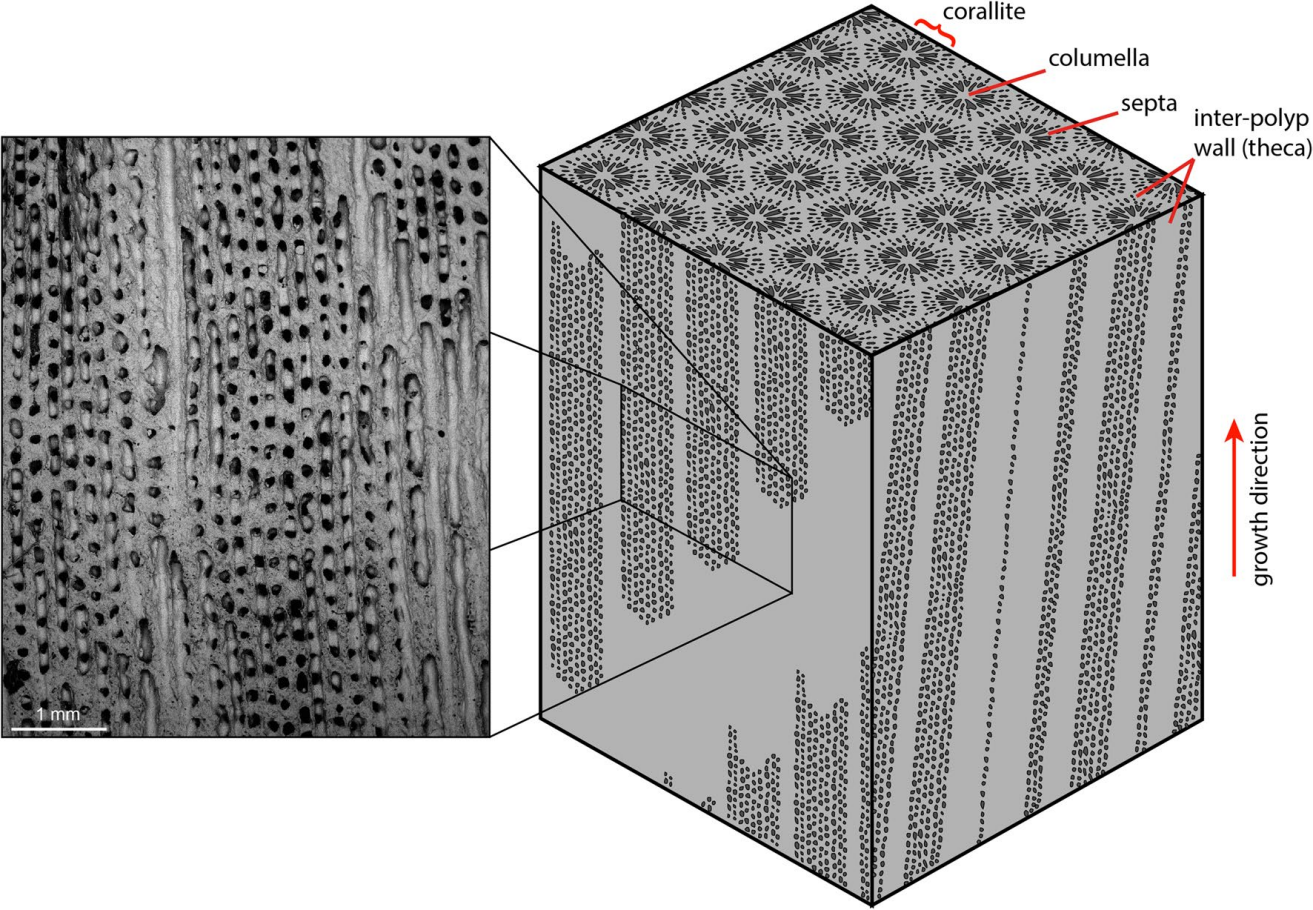
Schematic showing the complex three-dimensional structure of colonial coral skeletal material (in this case *Siderastrea siderea*). Coral polyps make up quasi-cylindrical structures (stippled) surrounded by denser wall structures (grey). When sectioned skeletal features will dip into and out of the section plane, as they do not grow along a perfectly straight axis in all dimensions, therefore it is imperative that a detailed understanding the skeletal structure measured is known. Mapping a planar surface allows the substructure to be penetrated. The analysed area is shown in the inset.

Previous attempts to examine ECM composition have primarily used micro-electrodes^8^ or pH sensitive dyes^9^. While these approaches have revealed many key insights, they are by their nature invasive, requiring either the insertion of needle-like electrodes into the calcification space or the growth of the coral on glass slides to allow direct observation of the ECM with confocal microscopy. Incisions are made to insert the micro-electrodes in the coral animal or via its oral orifice, which can adversely affect the organism and likely change the composition of the calcification space^10^, whilst growing the coral on a glass slide may disrupt some coral growth morphology and in turn impact the calcification process. In addition, both methods only provide snapshots of information for corals grown in laboratory conditions. The boron isotopic composition of the skeleton has a proven track record as a sensitive tracer of the pH of this calcifying fluid (pH_cf_) at the moment the skeleton is precipitated^11^, and when combined with ECM fluid [CO_3_^2−^] estimated from B/Ca^12^, can further interrogate the nature of the internal carbonate system^13^. This boron-based approach is non-invasive (though it has to be utilised on carbonate which has already been precipitated), and it can also be applied to samples grown in the field under natural conditions and over longer temporal intervals. Furthermore, if sampling resolution is sufficiently controlled, the boron isotope-based methods can more readily provide information regarding the spatial variability of the carbonate system within the ECM in different parts of the coral skeleton. A key example of this is between the porous columella (here used to define the interior of the polyp) and the densely calcified thecal wall (Fig. 1), which appear to respond differently to environmental stress that may underpin the organism-level response to environmental change^14,15^.

Previous boron isotope records of the pH_cf_ have typically (but not exclusively^16–18^) employed bulk sampling techniques such as micromilling which average across the various structural elements of the coral skeleton. These bulk approaches are restricted by the typical sample requirements of solution boron isotope analysis, i.e. > 0.5 mg of carbonate for a precision of < 0.3‰, which makes truly sampling discrete skeletal components in most species extremely challenging. Further complications arise from the geometry of coral growth (Fig. 1) which forms a complex and anastomosing structure often passing through the plane of section. The extent to which these structural components will impact bulk sampling data varies by species, but some degree of component mixing during sampling with bulk approaches is highly likely in most cases, and this will include pre-existing calibration studies which are critical to studies employing the boron isotope-pH proxy^19–24^. Secondary Ionisation Mass Spectrometry (SIMS) with a spatial resolution down to 10 µm, can overcome many of these spatial issues, but at the cost of precision and sample throughput (i.e. analyses are time consuming only allowing the examination of small analysis areas) that can limit the applicability of this technique with current methodologies^25–27^. In species with small-scale structure (e.g. *Porities* spp.), bulk sampling is perhaps more likely to provide a good mean value as the effective mixing of components will be more likely on the scale of a 0.5–1 mm drill-bit.

New analytical techniques now allow accurate in situ measurements of δ^11^B constrained to the fine scale structure of coral skeletons by employing laser ablation MC-ICP-MS^28–30^. Alongside elemental analysis by laser ablation ICP-MS^31^, such an approach is able to explore the spatial variation in boron isotopic composition and boron concentration (alongside other key element ratios) across the various skeletal components, ultimately enabling the construction of two-dimensional maps exploring the full carbonate system of the ECM. Here we apply this laser ablation methodology along with well-constrained bulk sampling methods for the first time to a tropical, reef-building, coral *Siderastrea siderea,* covering an ~ 18 month period of growth from ~ June 2006 to ~ January 2008. These analyses allow a reconstruction of the full carbonate system of the ECM from the skeleton at an unprecedented temporal and spatial resolution, permitting an examination of the influence of the carbonate system in the ECM on skeleton construction.

## Results

### Solution and laser ablation boron isotope data

**Figure 2:**
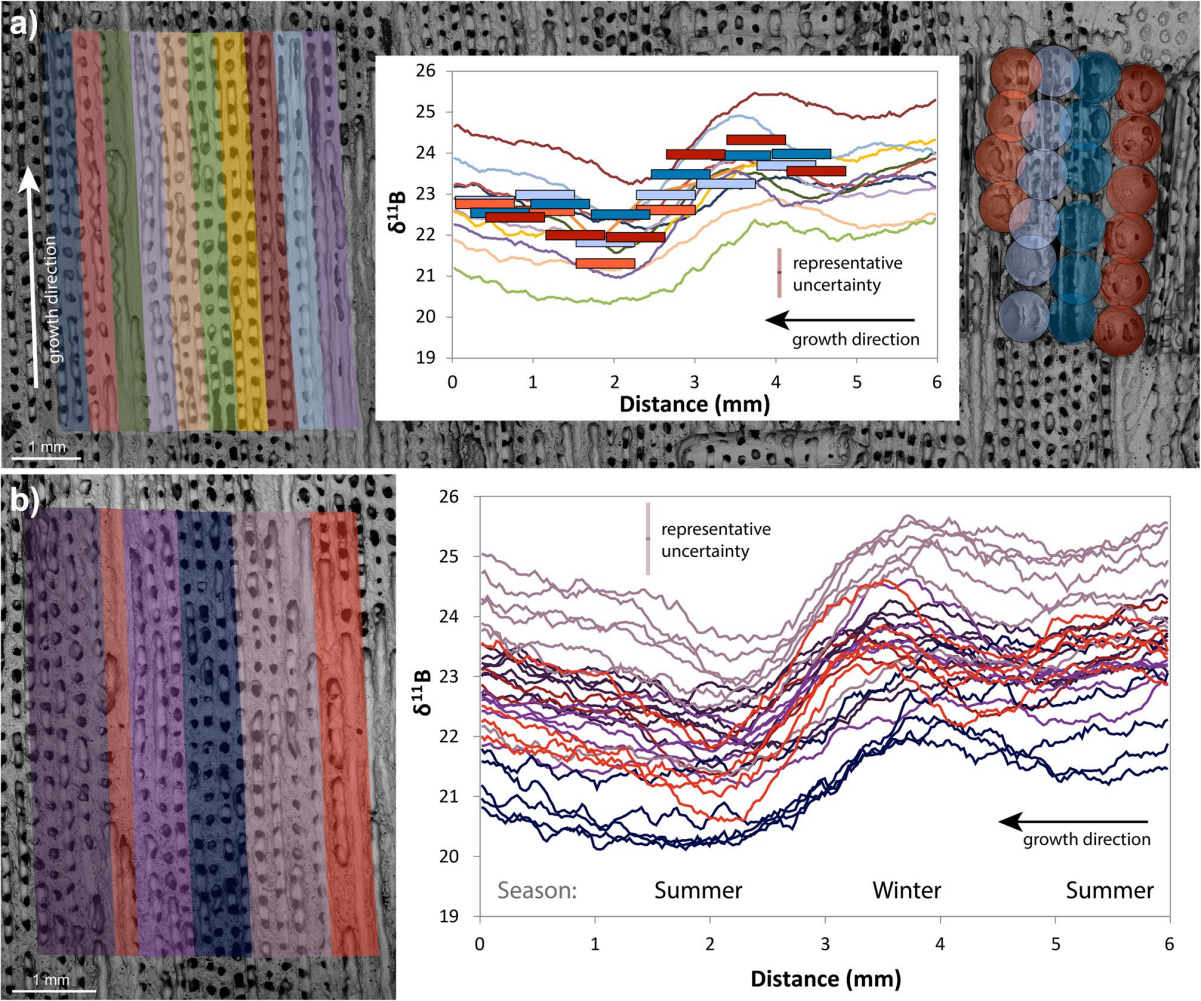
(**a**) SEM image of coral with: (left) traces of LA tracks grouped into sets of three (~ 450 µm wide from 150 µm width rasters), and (right) micro drilled sampling holes for solution analysis (500 µm width drill-bit) where red shows samples predominantly centred on the theca wall and the blue shows samples predominantly located within the columella of the polyp, where the radial septa join. The central plot shows corresponding δ^11^B data from LA (40-point running average) and solution methods. Solution boxes are sized to show 2σ uncertainties as box height and drill bit size as x-axis length, and data for both solution and LA are plotted as per the colours on the SEM image. Solution and laser data agree well within the different structural components, with the LA data showing higher variance as expected from the larger area and finer detail captured. The left side of the plot is the bottom of the SEM image, and the right side is the top. (**b**) Laser ablated transects (right, 40-point running averages) of δ^11^B from different skeletal elements (left) of same area of coral*.* Red colours indicate transects visually identified as predominantly theca wall, these show a clear and sharp annual cycle. Dark blue indicates the area around the columella, which shows a muted annual cycle and a lower average value. Purple colours indicate mixed transects where both distal columella, septa and theca wall edges are measured. Note: the seasonal cycle is visible in each group and in the solution data and does not vary widely in amplitude (see Figure S1). X-axis as above, representative uncertainty for LA data is shown in both plots.

Solution and laser ablation (LA) analyses were carried out on coeval polyps of the same colony of *Siderastrea siderea* (Fig. 2) in order to verify the accuracy of the laser ablation approach followed*.* Samples of the finest possible resolution were micromilled (~ 500 µm diameter = 0.2–0.5 mg) and analysed by solution, and these were compared to 30 linear transects covering an approximately equal area measured by LA. For further details see “Materials and methods” section.

The solution data (supplementary data 1) show a relatively smooth annual cycle of δ^11^B of ~ 2 ± 0.9‰ (2SD) ranging from 21.5 to 24.5‰ (red and blue boxes in centre plot and circles in right side SEM image, Fig. 2a). The columella dominated samples show a more muted annual cycle (22.5–24‰; blue shaded boxes and circles, Fig. 2) than the wall dominated samples which span the full range of variability (red boxes and circles). These data clearly highlight the importance of skeletal component in determining δ^11^B, given that there is > 1‰ difference in δ^11^B between the macro-structural elements of the polyp across a given temporal horizon (Fig. 2), here assumed to be approximately perpendicular to the growth axis.

To facilitate a better comparison to the solution data, the LA data in Fig. 2 were smoothed with a 40-point moving window (supplementary data 2). This treatment also reveals a broad annual cycle in all the tracks analysed, albeit with a slightly higher variance in comparison to the solution data (19.2–26.6‰; 5th and 95th percentiles for laser data, compared to 21.5–24.5‰ for solution data; Fig. 2), which is to be expected considering the higher initial resolution and the different resolution in the z-direction (~ 5 µm vs. ~ 500 µm for the LA and solution data respectively). The average amplitude from peak to trough along each individual track is 3.4 ± 1.2‰ (2SD, Figures S1 and S2), which is similar to the solution data, but there is no significant deviation from this amplitude between skeletal components. However, the average values of the tracks themselves varies from 21.2 to 24.5‰, a difference of 3.3‰ (Figure S1). There is a strong visual correlation between the mean of each track and the structural component analysed, with higher δ^11^B associated with components of the denser theca walls relative to the more porous internal parts of the polyp and columella (Fig. 2). We note that allocation of the ablated area to theca walls and columella is complicated by the structure beneath the plane of section that is visible on the SEM image, and that this may explain the highest δ^11^B in the columella closest to the visible walls (e.g. Fig. 1). An overall trend to decreased δ^11^B values of ~ 1‰ per year is visible across the analysed section for both analytical methods (i.e. lower δ^11^B towards the top of the coral piece). These data are also consistent with the low resolution annually averaged measurements of Fowell et al.^32^ from the same coral in terms of both the mean values and the apparent multiannual trend towards lower δ^11^B values from 2006 to 2008.

Over coeval horizons the LA and solution datasets show overlapping values and cyclicity (Fig. 2) that are consistent with previous comparisons^28^. This indicates that the two methodologies, as well as the δ^11^B-systematics in the two individual polyps from the colony analysed, are consistent. Indeed, the average of the solution data is 22.92 ± 1.58‰, compared to the laser average of 22.93 ± 3.29‰ (both at 2SD), with the increased variance of the LA data likely due to the capturing of finer scale details. Overall, the agreement between the two analytical methods is very good, which adds confidence to the utility of in situ LA as a means to improve our understanding of coral microstructure at a spatial scale unachievable by micromilling and solution based methods.

### Spatial patterns from mapping of δ^11^B and elemental ratios

**Figure 3:**
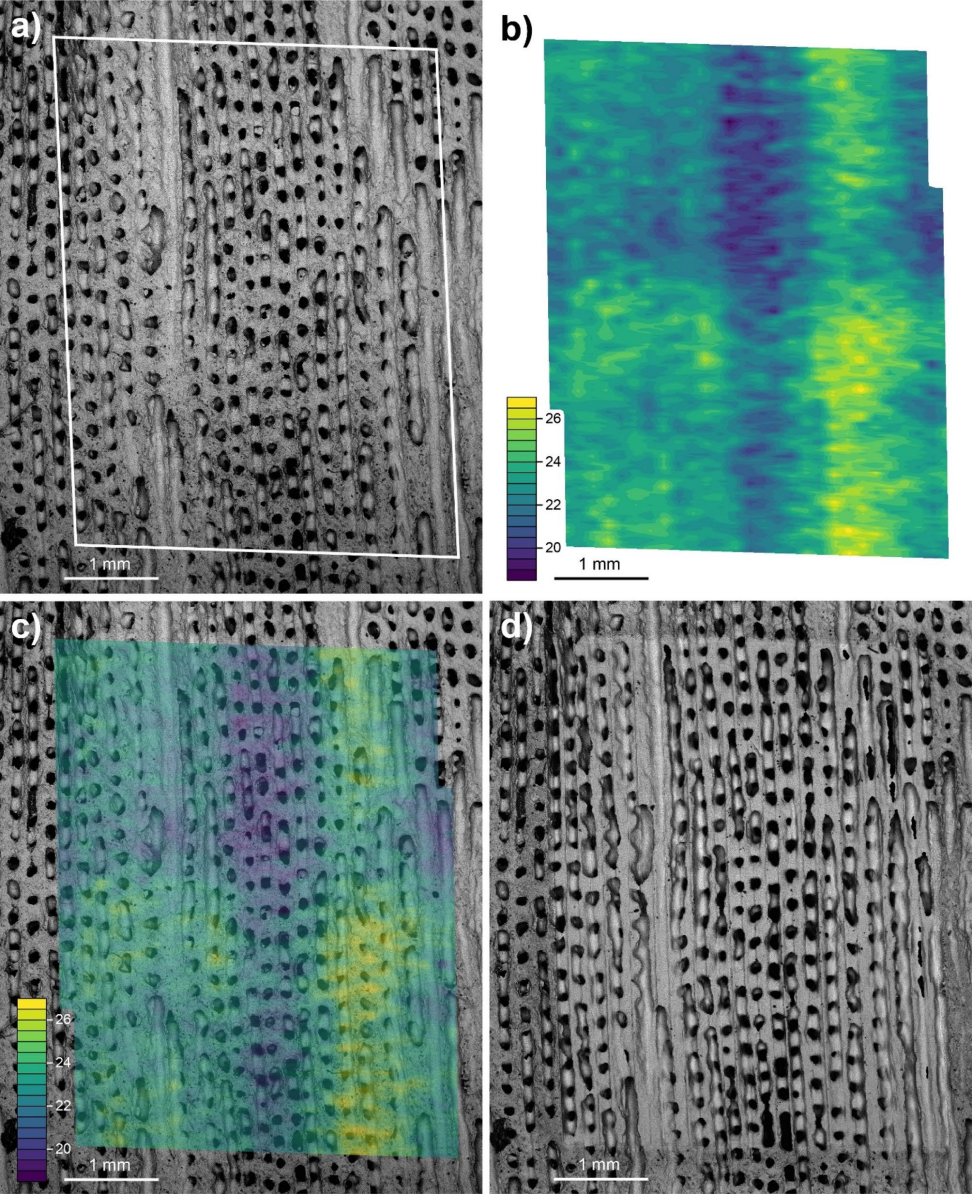
(**a**) Pre-ablation SEM image of the coral sector analysed by LA; (**b**) δ^11^B map of coral area, where yellow colours show high δ^11^B values (> 25‰), green colours show intermediary δ^11^B values (c.21–25‰) and blue colours show low δ^11^B values (< 21‰); (**c**) Overlay of δ^11^B map and SEM image, the values clearly match the structural elements of the coral with high values being confined to the polyp walls and low values to the columella. Sub-annual variability of ~ 1‰ is visible in the wall edges which may correspond to the dissepiments (not-visible). The two walls analysed show very similar trends but at different absolute values implying a potential 3-dimensional component to the analyses. (**d**) Post-ablation SEM image of the coral area ablated.

Given a knowledge of the X and Y locations, the unsmoothed LA data can be used to produce high resolution 2D maps of the analysed specimen (Fig. 3; see “Materials and methods” section), which in this instance provides an unparalleled insight into the nature of the relationship between coral macro-structure and the calcification processes. The higher spatial resolution enables a better measurement of the true variance of the δ^11^B within the skeleton, allowing the seasonal (and sub-seasonal) changes to be fully resolved rather than aliased by lower sampling resolution (e.g. unsmoothed the LA data shows a 7.4‰ 95th–5th percentile range compared with 2.9‰, for solution). In the 2D δ^11^B map shown in Fig. 3, the broad structural variations identified by the solution data, i.e. higher δ^11^B in the theca walls relative to the columella, are clearly illustrated. Moreover, the structure can be further refined within these two major structural divisions to identify small-scale features within the columella and theca walls. For instance, within the LA δ^11^B maps (Fig. 3) a small scale periodicity to the δ^11^B can also be seen, perhaps most easily at the interface of the wall and columella (see Fig. 3b). These variations are only ~ 1‰ which is small relative to the analytical uncertainty (typically ~ 0.6‰ 2SD based on the external reproducibility of the in-house coral reference material PS69/318-1) and we thus limit our interpretation of these features. They do, however, appear to have an occurrence frequency of 10–12 per year close to the lunar month frequency and therefore may be related to monthly aragonite dissepiment formation^33–35^.

**Figure 4:**
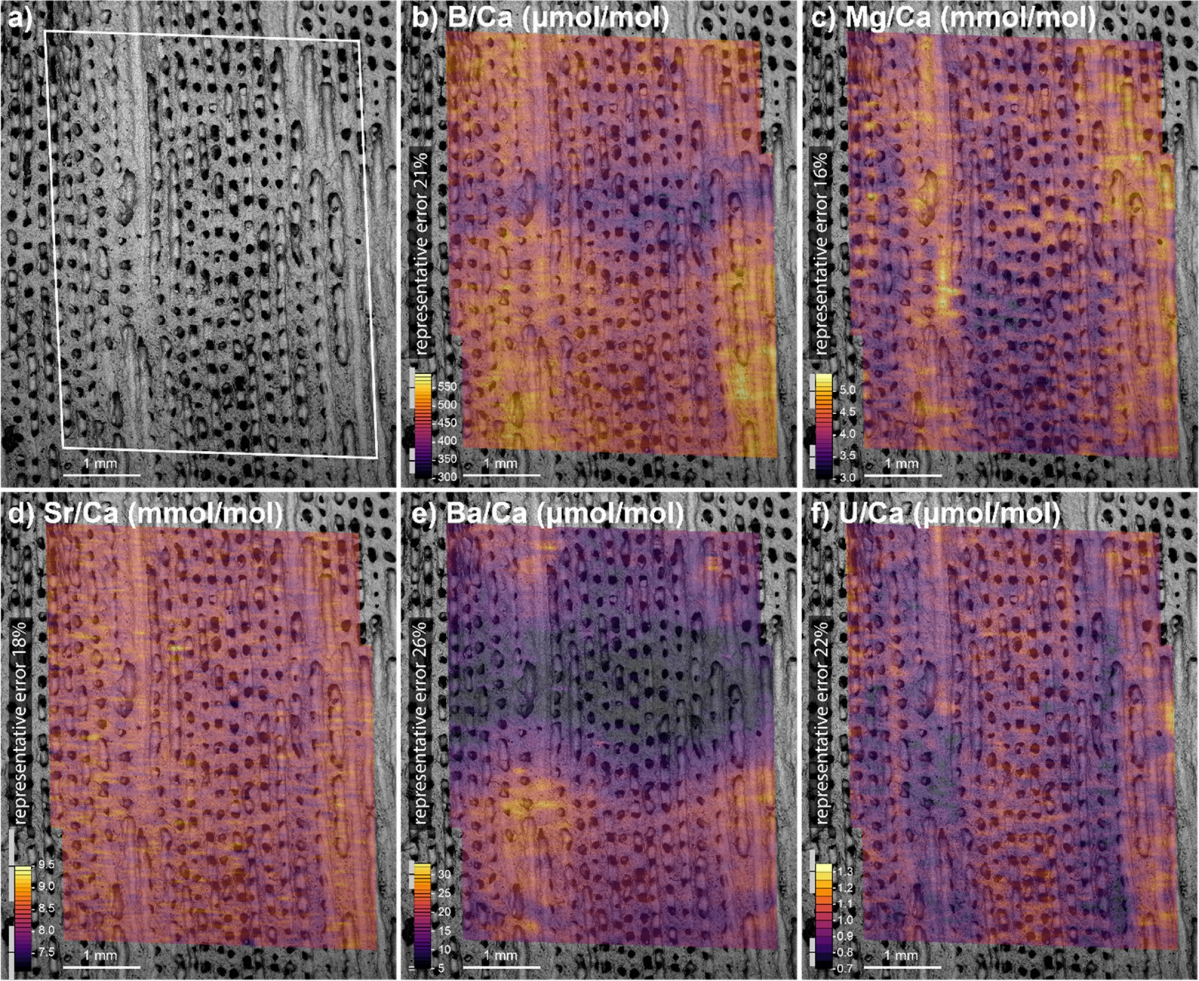
(**a**) Pre-ablation SEM image of the coral area analysed by LA, note the element ratio plots were analysed prior to the δ^11^B analyses as they utilise less material. The following plots are mapped onto the SEM image with high values shown in yellow colours and low values in purple: (**b**) B/Ca in µmol/mol ranging from < 350 to > 550 µmol/mol, B/Ca has a strong seasonal variation associated with changes to the internal carbonate system of the coral; (**c**) Mg/Ca in mmol/mol ranging from < 3.5 to > 5.0 mmol/mol; (**d**) Sr/Ca in mmol/mol ranging from < 8.0 to > 9.0 mmol/mol; (**e**) Ba/Ca in µmol/mol ranging from < 10 to > 30 µmol/mol, the Ba/Ca shows a strong seasonal cycle associated with riverine input, we note the visible undulation to the growth horizon as also seen in the B/Ca data showing the gross morphology of a coral polyp with raised theca walls and a depressed central polyp at any given time-step; (**f**) U/Ca in μmol/mol ranging from < 0.8 to > 1.1 µmol/mol. All trace and major element cations are higher concentration in the theca walls, with the exception of uranium (panel **f**). Uncertainties in the element ratios are shown as representative error bars at the top and bottom of the colour bars, for further information see Laser Ablation ICP-MS section in the methods.

The co-located 2D maps of skeletal trace element composition show that the theca walls of the polyps analysed have elevated δ^11^B, B/Ca, Mg/Ca, Sr/Ca, Ba/Ca relative to the centre of the polyp, and are depleted in U/Ca (Figs. 3 and 4). Like δ^11^B, many of the trace element ratios also show a seasonal cycle (i.e. 1.5 cycles over the 18 months of growth analysed). For δ^11^B, Mg/Ca and U/Ca the average change across the coral components (x-direction) is larger than that found along the growth axis (y-direction, see Table S3). In B/Ca, Sr/Ca and Ba/Ca however, temporal/environmental variability dominates over structural differences (see Table S1). This finding cautions the use of poorly resolved or bulk sampling strategies as a way to recover environmental data from coral aragonite, as this approach risks being unduly impacted by variability relating to coral macro-structure. This will be of particular importance in coral with large-scale macro-structure like *Siderastrea siderea*.

**Figure 5:**
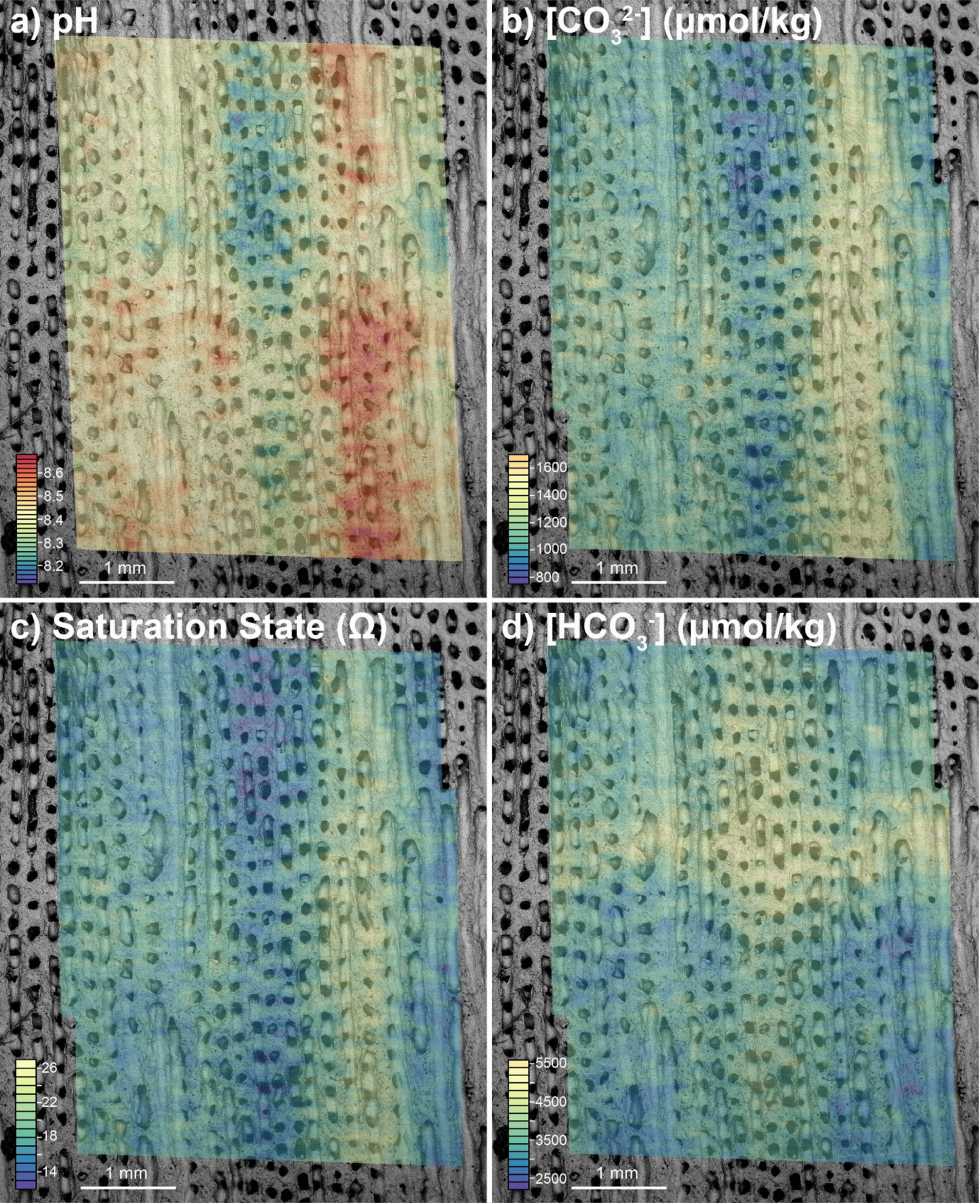
Four plots showing carbonate system parameters recovered from the calcifying fluid: (**a**) pH_cf_ calculated from δ^11^B, as is clear from the raw data the pH is elevated in the wall compared to the columella, though both show an annual cycle and sub-annual features are visible within the walls; (**b**) carbonate ion [CO_3_^2−^]_cf_ calculated from δ^11^B and B/Ca, similar to pH_cf_, carbonate ion is elevated in the walls and shows an annual cycle; (**c**) aragonite saturation state calculated using δ^11^B and B/Ca data, as expected from pH_cf_ and [CO_3_^2−^]_cf_ the saturation state is higher in the denser material of the walls, and tending towards seawater values at the centre of calcification; (**d**) bicarbonate (µmol/kg) calculated from δ^11^B and B/Ca. For further details see “Materials and methods” section.

In this study, we concentrate on the internal carbonate system as revealed by a combination of δ^11^B and B/Ca (see below), but we note that variations in some elements are clearly strongly related to variability in the carbonate system of the ECM. For instance, bulk sampling has revealed a negative relationship between coral U/Ca and pH^36^ and this is also replicated here between the wall and columella of a single polyp (e.g. comparing Fig. 4f with Fig. 5a).

### Coral calcification

Figure 5 shows 2D maps of the carbonate system variables pH, [CO_3_^2−^], aragonite saturation state in the calcifying fluid (Ω_cf_), and [HCO_3_^−^] calculated following established methodologies^12,13^ from δ^11^B and B/Ca data (as well as calcium, temperature, salinity and pressure, which are kept invariant for this study as their impact on the calculations is minor, see “Materials and methods” section for details). The calculated carbonate system parameters indicated pH, Ω_cf_, and carbonate ion ([CO_3_^2−^]) were higher in the ECM that deposited the theca walls, while [HCO_3_^−^] and thus DIC (as [HCO_3_^−^] dominates DIC at these pH values) were higher in the columella. There is an annual cycle in all structural elements, where the minimum pH in the theca walls (summer) is similar to the maximum in the columella (winter), this may be related to the difference in seasonal skeletal density. Centres of calcification (COC) may account for some of the higher variability resolved in the LA data, however, they are likely to be smaller than our spatial resolution (~ 10 μm) suggesting their role in generating the patterns we observe here is minor. However, we note that COC can be characterised by low δ^11^B and U/Ca, and high Mg/Ca^16,37,38^, which is not consistent with the larger spatial pattern observed here (e.g. columella with low δ^11^B and Mg/Ca, and high U/Ca).

To further investigate the association of elemental and isotopic distribution with structure we compare greyscale values of the SEM image with similarly processed images of the LA-derived ECM carbonate system data (See “Imaging comparison to porosity”, Figure S2). As pores are characterised by darker greyscale values relative to densely mineralised areas, this acts as a qualitative link between macro-structure/skeletal density and ECM composition. With this treatment we find a weak relationship between δ^11^B and porosity (r^2^ = 0.21, *p* value = 0.18), confirming the visual relationship evident in Fig. 3 (albeit only significant at the ~ 80% level of confidence). Table 1 summarises the relationship between porosity and all the calculated carbonate system parameters. There is clearly a strong covariance between skeletal density and ECM composition, with the strongest and only statistically significant being the negative relationships between skeletal density and both [HCO_3_^-^] and DIC (Figure S3, r^2^ = 0.42 and 0.55 respectively, *p* values = 0.04 and 0.01 respectively; Table 1), thought to be the crucial carbonate variables for controlling calcification^39–42^. These correlations imply that the porous columella aragonite is associated with a lower degree of pH-upregulation and higher degree of [HCO_3_^−^] (and thus DIC) elevation in the ECM than involved in the construction of the less porous thecal wall. This may be facilitated either by enhanced activity of bicarbonate anion transporters or increased passive diffusion of CO_2_ into the ECM in the columella region relative to the theca walls^43–45^. Importantly, the higher pH, [CO_3_^2−^] and Ω_cf_ in the thecal walls favours stoichiometric aragonite precipitation, required for the denser calcification of this structure.

**Table 1:**
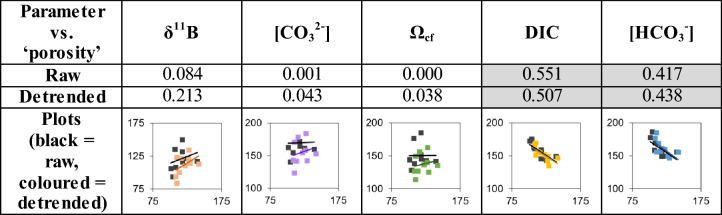
Correlation coefficient squared (r^2^) between ‘grey-scale value’, here a proxy for porosity, δ^11^B, and the reconstructed carbonate system variables as raw data and with linear trends removed. The strongest relationships are with DIC and [HCO_3_^−^] (and are negative), both in raw and detrended datasets. In the third row of the table are cross-plots with the ‘grey-scale value’ on the x-axis versus the carbonate system parameter on the y-axis. Black squares are raw data and coloured squares are detrended. For further details, see figure S2. The values in grey boxes indicate where the relationship is statistically significant (*p* < 0.05).

The most likely explanation for these patterns is that while the coral organism directly modifies the semi-isolated seawater reservoirs in the ECM to favour skeletal precipitation, it varies the degree of upregulation between skeletal components. The interior of the polyp is marked by lower pH, Ω_cf_, [CO_3_^2–^], leaving it closer in composition to that of ambient seawater, but with elevated [HCO_3_^−^] (and DIC) which is actively transported into the ECM^40^. Potentially the less dense skeleton in the columella region is the product of aragonite precipitation more heavily mediated by organic components, aided by the transport the bicarbonate ion, rather than from elevated pH as seen in the walls^3^. The difference in Ω_cf_ between wall (> 20) and columella (< 10) is particularly significant as elevated saturation state markedly favours precipitation of new crystals and enables dense growth of the organism in the walls^6,9^ (Fig. 5). These results show that the coral ECM is not a spatially homogenous fluid, but varies between skeletal components which in turn impacts skeletal porosity; denser skeleton is associated with higher pH and [CO_3_^2−^] and the more porous columella with elevated DIC and [HCO_3_^−^] (~ 2 times above seawater).

Taken together, these findings show that the calcification process in *Siderastrea siderea* is highly dependent on which part of the skeleton is being precipitated, meaning that the coral organism potentially has access to a diverse “biomineralisation tool kit” which may lead to different strategies being employed in aragonite precipitation in different parts of the skeleton. This has mechanistic implications for results seen in laboratory experiments where lowered seawater pH commonly leads to a more porous skeleton^3,15^. Indeed, Horvath et al.^14^ note that in *Siderastrea siderea* much of the decline in calcification associated with decreasing seawater pH was the result of less in-filling of the corallite, i.e. that the columella became more porous at low seawater pH. Our results suggest that this behaviour may be the result of either: (i) the lower pH_cf_ and Ω_cf_ upregulation of the columella region causing calcification in that region to be impacted by declining seawater saturation state to a greater extent than the theca wall; and/or (ii) the higher pH_cf_ and Ω_cf_ seen in the theca walls may result in a greater relative impact of acidification on the carbonate system in the ECM of the thecal wall, reducing wall calcification to a greater extent than columella calcification, decreasing overall density and leading to a particular calyx being made up of more porous columella than dense wall. Regardless, variability in the nature of the tools employed by the coral for skeletal construction appear to dictate the response of the skeletal phenotype at least to this particular environmental stress.

### Repercussions for using δ^11^B to reconstruct past seawater pH variability

Beyond the implications for biomineralisation, the findings from our detailed mapping of the isotopic and chemical composition of the skeleton of *Siderastrea siderea* has wide-ranging repercussions on sampling for the reconstruction of past environmental variables. We find that theca wall samples, traditionally identified for easy sampling and large signal-to-noise ratios in geochemical studies^46^ are furthest removed from the pH of ambient ocean water. The central polyp, which has the same amplitude of annual cycle as the theca wall, is characterised by a pH_cf_ that is closest to pH_sw_. Variable mixing of structural components during sampling therefore has the potential to influence the resulting reconstructed pH signal, as the variation across the polyp is similar in scale in *Siderastrea siderea* at least to the pH variability associated with an annual cycle (Table S1). Further calibration of the δ^11^B-pH proxy is needed focusing on these structural elements, and the creation of multipolyp maps such as these are recommended to aid in the interpretation of laser ablation δ^11^B data in such future studies. Across almost the entire polyp structure, the amplitude of change (peak-to-trough) in an annual cycle is constant, barring the very centre of the polyp, which is also evident in the solution data. This shows that existing δ^11^B-derived pH records are likely to be correct regardless of component measured, as long as mixing proportions are approximately constant.

## Conclusions

We have shown that using laser ablation mapping techniques it is possible to fully constrain the carbonate system of the internal calcifying fluid of the coral *Siderastrea siderea* at < 200 µm scale or sub-monthly temporal resolution. The detailed structure of the coral shows sub-annual variation that cannot be identified by traditional microdrilling techniques and, notably, also picks apart the different pH and carbonate parameters of the calcifying fluid which are used to precipitate different skeletal components.

Higher pH is maintained annually in the denser theca walls while the centre of the polyp shows the lowest pH and Ω_cf_ and is thus the least modified from ambient seawater (although still with elevated DIC and [HCO_3_^−^] to twice ambient seawater concentrations). We propose that these variations in the extent of pH and Ω_cf_ modification leads to ocean acidification having a variable effect on each structural component. Thus providing mechanistic insights into the causes of the observation^14^ that ocean acidification leads to less infilling of the corallite of *Siderastrea siderea*. Mapping the chemical and isotopic composition of coral skeletons in the fashion described here therefore provides a unique window into the processes occurring within the (sub-)micron-sized ECM and offers a new means to probe the effect of environmental change on coral calcification.

## Materials and methods

### *Siderastrea siderea* sampling

In December 2009, a *Siderastrea siderea* coral was cored (sample BR-06) from ~ 5 m water depth on the back reef of the southern Belize portion of the Mesoamerican Barrier Reef System (16.14045° N, 88.26015° W) using a 2 horse power hand-held pneumatic air drill with a custom hollow extension rod and wet diamond core bit^32,47,48^). A 5 mm thick slab was removed from the core and divided into 80 mm sections using a diamond-tipped tile saw, then individual sections were polished using a diamond plate grinder followed by silicon carbide grinding paper and mounted onto glass slides. The uppermost portion of this core was used for the present study, correlating to skeletal growth between ~ June 2006 and ~ January 2008 (i.e., in and around 2007).

### Solution MC-ICP-MS

All geochemical analyses were performed at the University of Southampton. Solution δ^11^B analysis was performed using a Thermo Scientific Neptune MC-ICP mass spectrometer (Thermo Fisher Scientific, Waltham, MA, USA) following previously published procedures^49,50^. Twenty-two samples (0.2–0.5 mg) were micro drilled in a grid formation from the *Siderastrea siderea* slab, encompassing the full width of a single corallite (i.e., theca wall to theca wall) and approximately 18 months of growth, using a New Wave Research Micromill and a 500 μm drill-bit. The individual holes are 500 µm in depth. Samples were oxidatively cleaned in 1% H_2_O_2_ at 80 °C for 5 min and leached in 0.0005 M HNO_3_ prior to being dissolved in ∼ 0.15 M HNO_3_. Boron was separated from the carbonate matrix by ion-exchange chromatography using 20 μl Teflon columns containing Amberlite IRA743 resin, alongside a coral standard (Japanese Coralline *Porites* [JCp-1]). Mass bias corrections were applied by sample-standard bracketing using boron isotope standard NIST SRM951. A total procedural blank correction was applied based on analysis of the blank from the chemical separation stage, which was also used to correct the JCp-1 standard. Solution concentrations ranged from 5 to 40 ppb B and uncertainty (2σ) ranges from ± 0.6 to 0.2‰, respectively. The data are presented in supplementary data 1.

### Laser ablation MC-ICP-MS

Laser ablation δ^11^B isotope analysis was performed using a Thermo Scientific Neptune Plus MC-ICP mass spectrometer coupled to an Elemental Scientific Lasers (Bozeman, MT, USA) NWR193 excimer laser ablation system with a TwoVol2 ablation chamber, housed in the Geochemistry laboratory at the University of Southampton. Prior to laser ablation MC-ICP-MS the *Siderastrea siderea* sample was removed from the glass slide and cleaned in 20% H_2_O_2_ to remove remnant organic material, then ultrasonicated for 10 min and rinsed three times in 18.2 MΩ cm (ultrapure) water.

LA-MC-ICP-MS analysis broadly followed protocols published by Standish et al.^28^. Boron isotopes were measured on the L3 and H3 Faraday cups installed with 10^12^ Ω resistors. Typical operating conditions are outlined in Table S2. Prior to data collection, the samples and standards were pre-ablated to remove any surface contamination. Data were collected in static mode using integrations of 2.194 s and a spot size of 140 µm. Analyses of reference materials integrated 100 cycles. Dynamic blank corrections were applied cycle by cycle assuming a linear relationship between the preceding and succeeding blank measurements. Instrumental mass bias was corrected by sample-standard bracketing with glass reference material NIST SRM610 and the isotope composition published by le Roux et al.^51^ and Standish et al.^28^. Matrix interference from scattered Ca ions on ^10^B were corrected for using the log-relationship between δ^11^B inaccuracy and ^11^B/Ca_interference_ for carbonate reference materials JCp-1 (*Porites sp.* coral) and JCt-1 (biogenic aragonite *Tridacna gigas*)^52^, where the Ca_interference_ was measured at *m/z* of 10.10 using the L2 Faraday cup (also installed with a 10^12^ Ω resistor). Data were screened and those cycles falling outside of 3 SD of the mean were removed. The data are presented in Table S3.

Internal reference material PS69/318-1 (Table S3), a fragment of a deep sea, cold water, calcitic Scleraxonian octocoral, was ablated during the analytical session (n = 3) giving a mean δ^11^B value of 14.04 ± 0.60‰ (2 SD), consistent with a solution MC-ICP-MS measurement of 13.83 ± 0.29‰ (2σ). Thirty adjacent 140 μm wide transects (circular laser beam 140 μm in diameter) of the *Siderastrea siderea* were ablated equating to an area of ~ 4.2 by 6 mm that encompassed approximately 1.5 corallites and corresponding to the same period of growth as the solution MC-ICP-MS analyses. The approximate depth of the ablated trenches was ~ 5 µm.

### Topographic impact on laser data

We highlight a difference between the δ^11^B signature of the coral depending on the degree of porosity, and there is a possibility that this variability relates to analytical artefacts produced when ablating the irregular and/or rough surface of the coral as the laser beam tracks across the porous skeleton, e.g. due to an instrumental fractionation of the ^11^B/^10^B or ^11^B/Ca_interference_. To exclude this possibility, synthetic ‘pores’ and trenches were created in a piece of NIST SRM612 (silicate glass reference material) to mimic the topography of the coral structure (see figure S4a). Five laser ablation transects were analysed over this area (Figure S4b) and the data processed in an identical fashion to the coral data. No discernable variation in the δ^11^B relating to this topography was detected, therefore implying surface topography has little control on δ^11^B measured by laser ablation. We further this analysis by investigating the Ca intensity in the ICP-MS data for our target section of *Siderastrea siderea* compared to B/Ca measured in the same session and the SEM image (Figure S5). We find the Ca intensity picks out the pores well (a vs. b) but this pattern is not replicated in the B/Ca, showing little impact from the porosity or topography. We attribute this to the normalisation to Ca and the lack of any significant fractionation of B to Ca due to sample topography. We also present Ca_interference_ (from MC-ICPMS measurements at 10.10 amu) vs. δ^11^B from four representative transects, two in the walls and two in the columella. We find no correlation of Ca_interference_ (which can be viewed as a proxy for the amount of sample material being ablated) with δ^11^B in any of the transects despite the clear reduction in intensity of the Ca-interference over the higher porosity columella (top blue plots vs. bottom red plots). These findings are in good agreement with the study of Thil et al.^53^ that also noted surface irregularities resulting from coral skeletal porosity have little or no impact on the accuracy of laser ablation δ^11^B.

### Laser ablation ICP-MS

Laser ablation trace element analysis was performed on a Thermo Scientific X-Series II Quadrupole ICP mass spectrometer coupled to an Elemental Scientific Lasers (Bozeman, MT, USA) NWR193 excimer laser ablation system with a TwoVol2 ablation chamber, housed in the Geochemistry laboratory at the University of Southampton. Typical operating conditions are outlined in Table S2. Prior to data collection, the standards were ablated to remove any surface contamination. An on-peak gas blank subtraction was performed using the mean of bracketing gas blank analyses. Raw elemental ratios were normalised against bracketing analyses of carbonate reference material JCp-1 and values^54^. Data were screened and those cycles falling outside of 3 SD of the mean were removed. Internal reference material PS69/318-1 was again run as a consistency standard, giving good accuracy and reproducibility (Table S3). Thirty adjacent 140 μm wide transects (square laser beams of 140 by 50 μm area) of the *Siderastrea siderea* were ablated covering the same area which had previously been ablated for δ^11^B isotope analysis. Uncertainty, which ranges from 16 to 26% on all ratios (2σ), was determined from whichever was the higher out of: the 2SD of repeat measurements of the deep sea coral reference material PS69/318-1 or the mode (to two significant figures) of a 15-point running mean of the entire element/Ca dataset, which captures reproducibility in the gridded data but without being unduly impacted by rapid environmental changes. Both of these are considered to be conservative approaches.

### Isotopic and elemental mapping

Despite the isotopic and trace element data being collected using the same laser conditions and scan speed, the different integration times for the different mass spectrometric techniques resulted in the δ^11^B and trace element line profiles having a different initial resolution (272 vs. 3311 data points per line). Each laser line for δ^11^B and trace elements was firstly subjected to a 3SD rejection to remove any outliers and then a simple moving average was used to smooth the data. The width of the moving average window was 5 points for the δ^11^B data and 15 points for the trace element data. The 30 separate smoothed laser lines were mapped onto an equal spaced grid using their X and Y spatial coordinates from Elemental Scientific Lasers (Bozeman, MT, USA) NWR193 excimer laser ablation system. The dimensions of the grid were governed by the resolution of the δ^11^B data and was constructed using the Raster package^55^ in R^56^ utilising the “filledcontour” function. The X–Y resolution of the images produced was 147 × 21 microns per pixel.

### Mapping of the carbonate system

Reference points were used to precisely align the δ^11^B and B/Ca grids, with the former resampled onto the grid of the latter. The combined data from δ^11^B (converted to pH as in Eq. 1) and B/Ca (Eq. 2) was then used to calculate the carbonate system (largely following DeCarlo et al.^12^ and Holcomb et al.^57^). In these calculations pK_B_ is assumed constant, i.e. we are not accounting for temperature. This assumption however, will only have small impact on these data as the seasonal temperature change in this region is 3 °C = 0.3‰ change in δ^11^B. The carbonate data were calculated using the values from the relevant raster grid and imaged using the Raster function in R^55,56^.

1 $$pH_{cf} = pK_{B}^{{}} - \log \left( { - \frac{{\delta^{11} B_{SW} - \delta^{11} B_{{{\text{CaCO}}_{3} }} }}{{\delta^{11} B_{SW} - \alpha_{B} \cdot \delta^{11} B_{{{\text{CaCO}}_{3} }} - 1000 \cdot \left( {\alpha_{B} - 1} \right)}}} \right)$$

where δ^11^B_sw_ = 39.61‰^58^, pK_B_ = 8.6 and the isotopic fractionation factor (α_B_) = 1.0272 Klochko et al.^59^.

To calculate [CO_3_^2−^] (in µmol/l) we used a simplified relationship between [CO_3_^2−^], borate ion ([B(OH)_4_^−^]_cf_ in µmol/l) and B/Ca (µmol/mol) fitted to the data of Holcomb et al.^57^:

2 $$\left[ {{\text{CO}}_{3}^{2 - } } \right]_{cf} = 0.00297 \times \left( {\frac{{\left[ {{\text{B}}\left( {{\text{OH}}} \right)_{4}^{ - } } \right]_{cf} }}{{\text{B/Ca}}} \times 10^{6} } \right)$$

where [B(OH)_4_^−^]_cf_ is calculated, following Dickson^60^ and using the seawater boron concentration from Lee et al.^61^, from:

$$\left[ {{\text{B}}\left( {{\text{OH}}} \right)_{4}^{ - } } \right]_{cf} = \frac{432.6}{{1 + \left[ {\text{H}} \right]^{ + } {/}K_{B} }}$$

Seawater [Ca] is kept constant at 10 mmol/kg and other carbonate system parameters were calculated following the equations in^12,62^.

### Imaging comparison to porosity

To compare the reconstructed carbonate system variables to porosity, we convert all the maps and the SEM image to 8-bit (0–255 value) greyscale in ImageJ^63^. In the SEM image grey scale value is primarily related to the porosity and therefore is a proxy for skeletal density (or amount of carbonate in a particular region). For all the carbonate variables, the colour schemes used indicate that lighter colours are associated with higher values (Figure S2). These greyscale values are averaged in 100 pixel-wide vertical bins to give an average for the various skeletal components (which run approximately vertically in our sectioned coral). We then cross plot the ‘porosity’ proxy values against each of the carbonate parameters both as raw data and with any long-term trend (which may be indicative of a long-term environmental shift) removed by subtraction (Figure S3). The cross-correlation values for each parameter are shown in Table 1, and the cross plots for DIC and [HCO_3_^−^] are shown in Figure S3.

## Supplementary information

Supplementary Information 1.

Supplementary Information 2.

Supplementary Information 3.
