# Supplementary material for: Mapping coral calcification strategies from in situ boron isotope and trace element measurements of the tropical coral Siderastrea siderea
Source: Sci Rep. 2021 Jan 12;11:472. doi: 10.1038/s41598-020-78778-1 (PMC7804963; doi:10.1038/s41598-020-78778-1)
Supplement: Supplementary file 3 — Supplementary Information 3. [file 41598_2020_78778_MOESM3_ESM.docx]

**Supplementary materials for “Mapping coral calcification strategies from in situ boron isotope and trace element measurements of the tropical coral *Siderastrea siderea”.***

T.B. Chalk^1,^*, C.D. Standish^1^, C. D’Angelo^1^, K. D. Castillo^2^, J. A. Milton^1^ and G.L. Foster^1^.

*correspondence to t.chalk@noc.soton.ac.uk.

^1^ Ocean and Earth Sciences, National Oceanography Centre Southampton, University of Southampton, Southampton, UK.

^2^ Marine Sciences, University of North Carolina at Chapel Hill, Chapel Hill, NC, USA.

**Supplementary materials**

**Tables and data**

**Solution data**

See supplementary book 1

**Laser data**

See supplementary book 2

**Table S1**. Lateral vs. vertical variability.

| Element/isotope ratio | X-direction mean 95th-5th %tile (2SD) [structural] | Y-direction mean 95th-5th %tile (2SD) [temporal] | Ratio Y/X |
| --- | --- | --- | --- |
| δ^11^B | 3.28 ‰ (0.92) | 2.11 ‰ (1.37) | 0.64 |
| B/Ca | 61.5 µmol/mol (38.9) | 93.5 µmol/mol (56.1) | 1.52 |
| Mg/Ca | 0.64 mmol/mol (0.41) | 0.42 mmol/mol (0.27) | 0.66 |
| Sr/Ca | 0.30 mmol/mol (0.19) | 0.55 mmol/mol (0.36) | 1.80 |
| Ba/Ca | 4.60 µmol/mol (4.23) | 12.8 µmol/mol (8.85) | 2.78 |
| U/Ca | 0.15 µmol/mol (0.10) | 0.10 µmol/mol (0.06) | 0.64 |

**Table S1**: Mean differences between the average change in X-direction (predominantly structural) per line and the Y-direction (temporal change), as shown by 95^th^–5^th^ percentile of each row or column. The 2SD of each group is shown in brackets. The ratio Y/X shows if the variable is predominantly controlled by structure (<1) or by temporal/environmental (>1) factors. Note: variability in the X direction also takes into account variation between multiple polyps.

**Table S2.** Typical operating conditions (Laser).

|  | **δ^11^B isotope analysis** | **Trace element analysis** |
| --- | --- | --- |
| **Instrument** |  | |
| Mass Spectrometer | Thermo Scientific Neptune Plus multi-collector inductively coupled plasma mass spectrometer | Thermo Scientific X-Series II Quadrupole inductively coupled plasma mass spectrometer |
| Laser Ablation System | Elemental Scientific Lasers NWR193 excimer laser ablation system with a TwoVol2 ablation chamber | Elemental Scientific Lasers NWR193 excimer laser ablation system with a TwoVol2 ablation chamber |
| RF Power | 1400 W | 1350 W |
| Cones | Nickel X skimmer and jet sample | Nickel XT skimmer and standard sample |
|  | | |
| **Gas Flows** |  | |
| Cooling Gas (argon) | 16 l min^-1^ | 13 l min^-1^ |
| Auxiliary Gas (argon) | 0.8 l min^-1^ | 0.8 l min^-1^ |
| Make-up gas (argon) | 1.0 l min^-1^ | 0.35–0.40 l min^-1^ |
| Ablation cell carrier gas (helium) | 0.8–1.0 l min^-1^ | 0.8–1.0 l min^-1^ |
| Additional Gas (nitrogen) | 0.001–0.006 l min^-1^ | 0.001–0.006 l min^-1^ |
|  | | |
| **Ablation Conditions** |  | |
| Laser power denisty | ~6 J cm^-2^ | ~6 J cm^-2^ |
| Laser repetition rate | 12 Hz | 5 Hz |
| Laser beam size | 100–140 μm diameter | 140 by 50 μm |
| Laser tracking speed | 10 μm s^-1^ | 10 μm s^-1^ |
| Ablation mode | Line | Line |

**Table S3.** Laser standard data

| **MC-ICP-MS and ICP-MS analyses of internal reference material deep sea coral PS69/318-1**  (Cold water calcitic Scleraxonian octocoral) | | | | | | |
| --- | --- | --- | --- | --- | --- | --- |
|  | δ^11^B | B/Ca (μmol/mol) | Mg/Ca (mmol/mol) | Sr/Ca (mmol/mol) | Ba/Ca (μmol/mol) | U/Ca (nmol/mol) |
| Solution | 13.83 ± 0.29 (2 SE) | 198 | 82.5 | 2.8 | 12.9 | 16.9 |
| Laser Ablation (± 2 SD) | 14.04 ± 0.6 | 223.4 ± 9.6 | 82.8 ± 1.4 | 2.9 ± 0.1 | 13.3 ± 1.5 | 16.2 ± 1.2 |

Solution values from Foster, et al. ^47^ for trace elements and Standish, et al. ^27^ for δ^11^B.

**Supplementary figures**


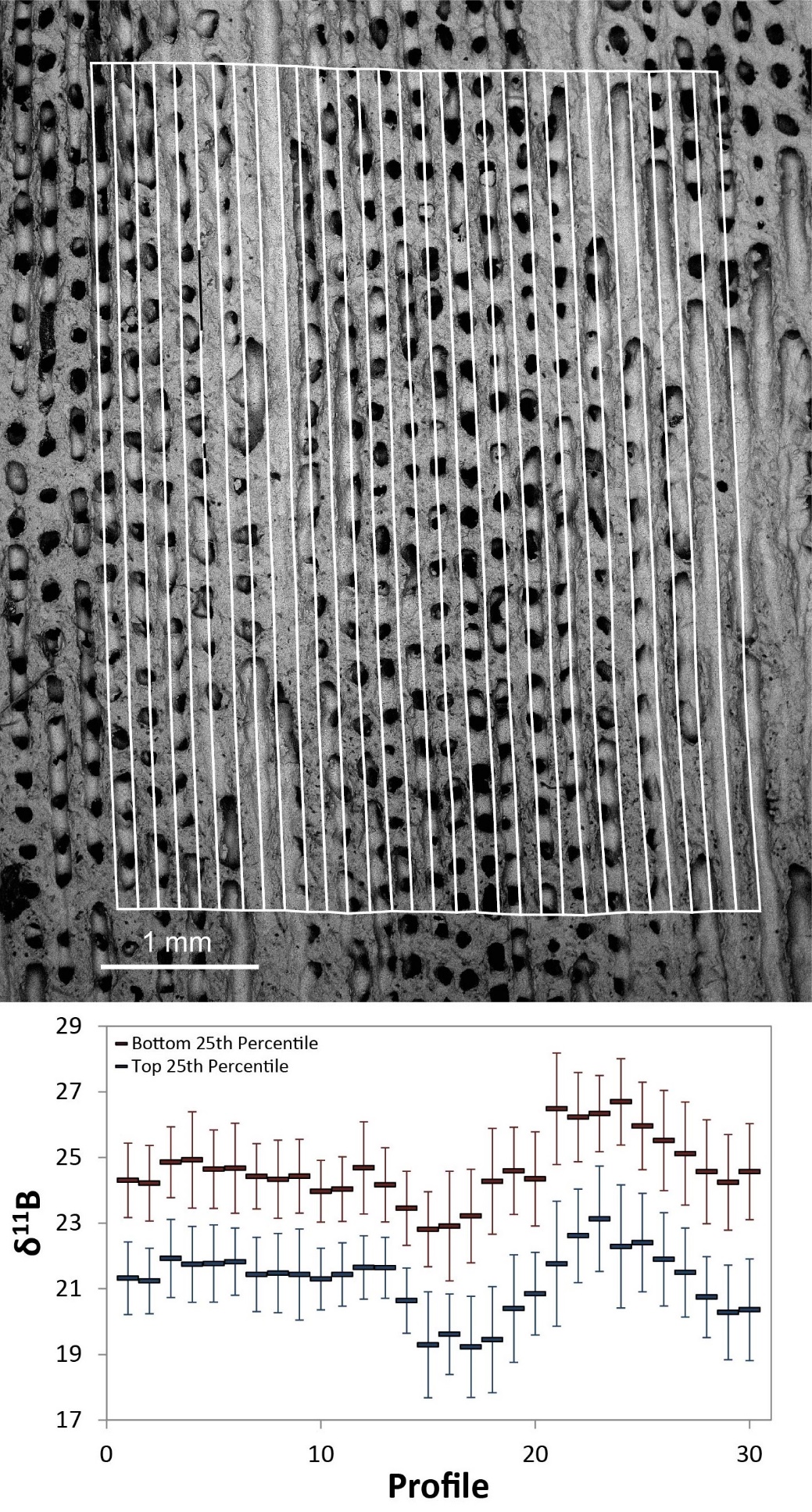


**Figure S1.** Top: Each transect line overlain on the SEM image of the coral specimen. Bottom: The top (red) and bottom (blue) 25 % percentile (± 2SD) of the δ^11^B values in each segment. Note that the range between top and bottom (i.e. cycle amplitude) is approximately equal and around 4 ‰ regardless of sampling zone. However, the absolute values change with the centre of calcification showing values of ~19–23 ‰ and the walls showing values of ~23–27 ‰.


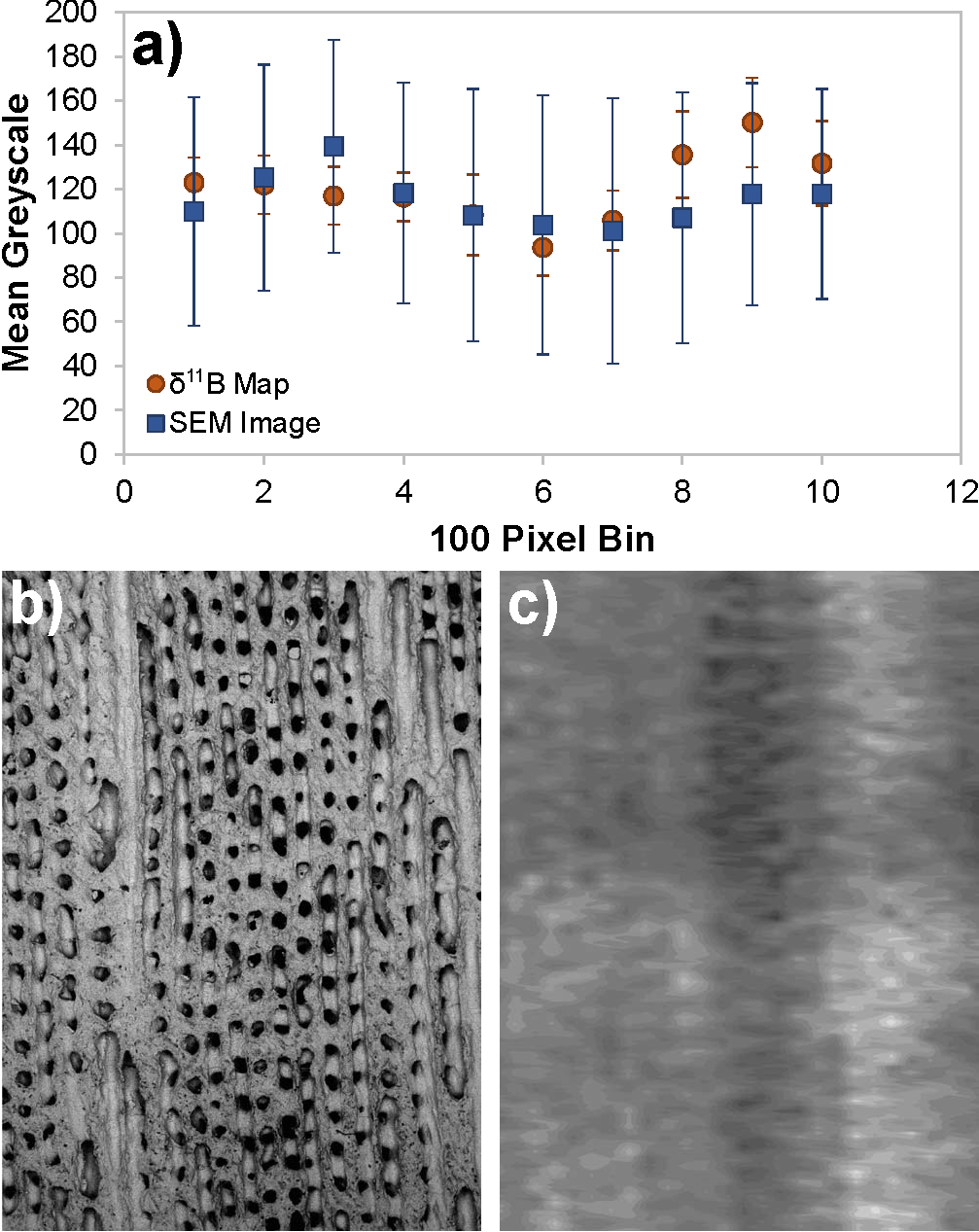


**Figure S2.** a) mean greyscale values of the coral SEM image (blue squares and panel b) and a greyscale version of the δ^11^B isotope map (orange circles and panel c). Images are divided into 100 pixel wide vertical bins for greyscale calculation. b) SEM greyscale image. c) greyscale δ^11^B plot.


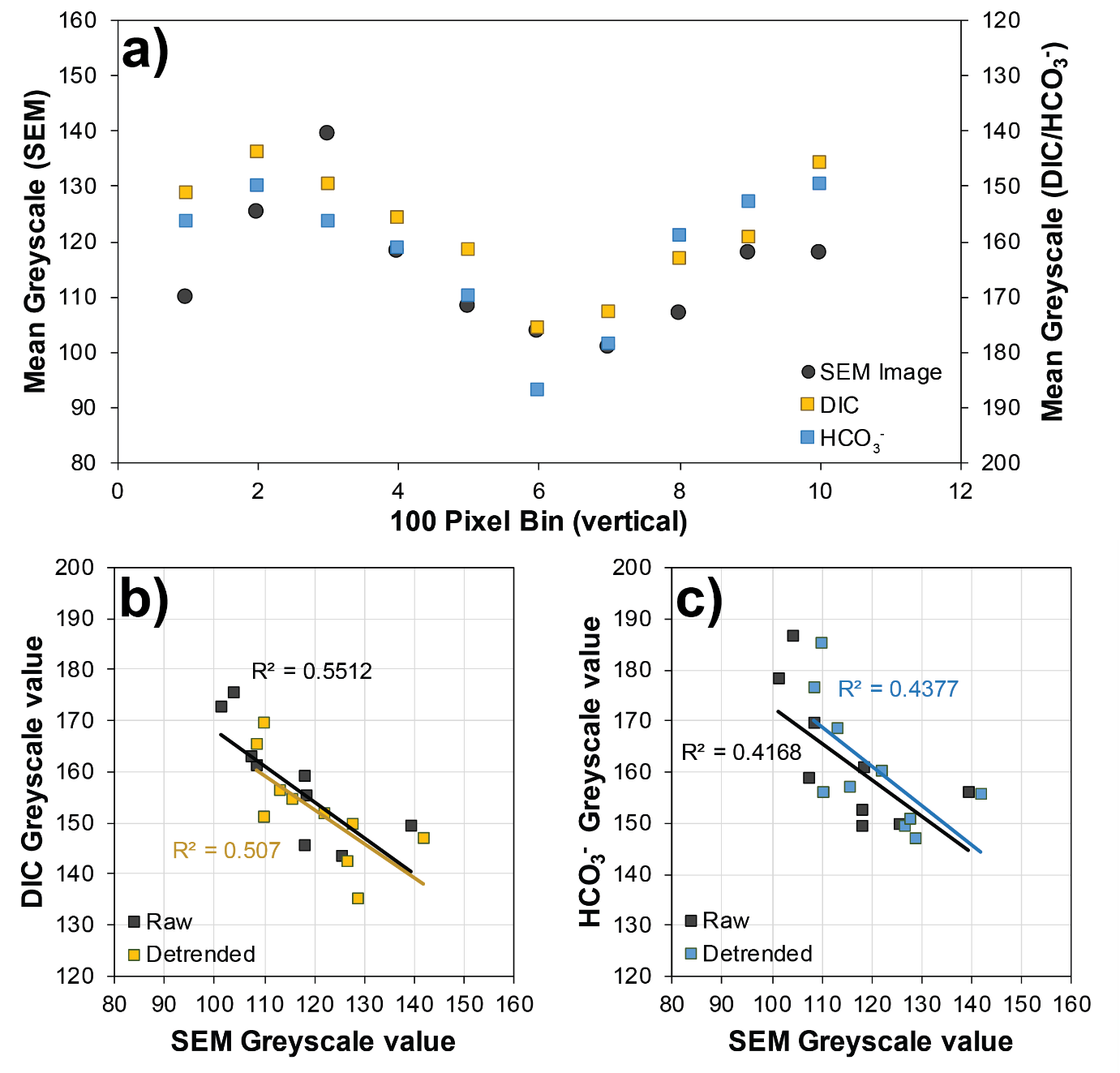


**Figure S3.** a) mean greyscale values of the coral SEM image (black circles) and a greyscale mean of version of the DIC and [HCO_3_^-^] maps (yellow and blue squares respectively), note the inverted axis for the carbonate variables. Constructed in the same way as figure S2. b) raw (black) and detrended (yellow) cross-plots comparing the SEM and DIC greyscale plots, shows a strong negative relationship between greyscale value (a proxy for density, increasing at higher greyscale values) and DIC (as reconstructed by δ^11^B and B/Ca). c) as b) but with raw (black) and detrended (blue) cross-plots comparing the SEM and [HCO_3_^-^] greyscale plots, which shows a slightly weaker relationship than DIC.


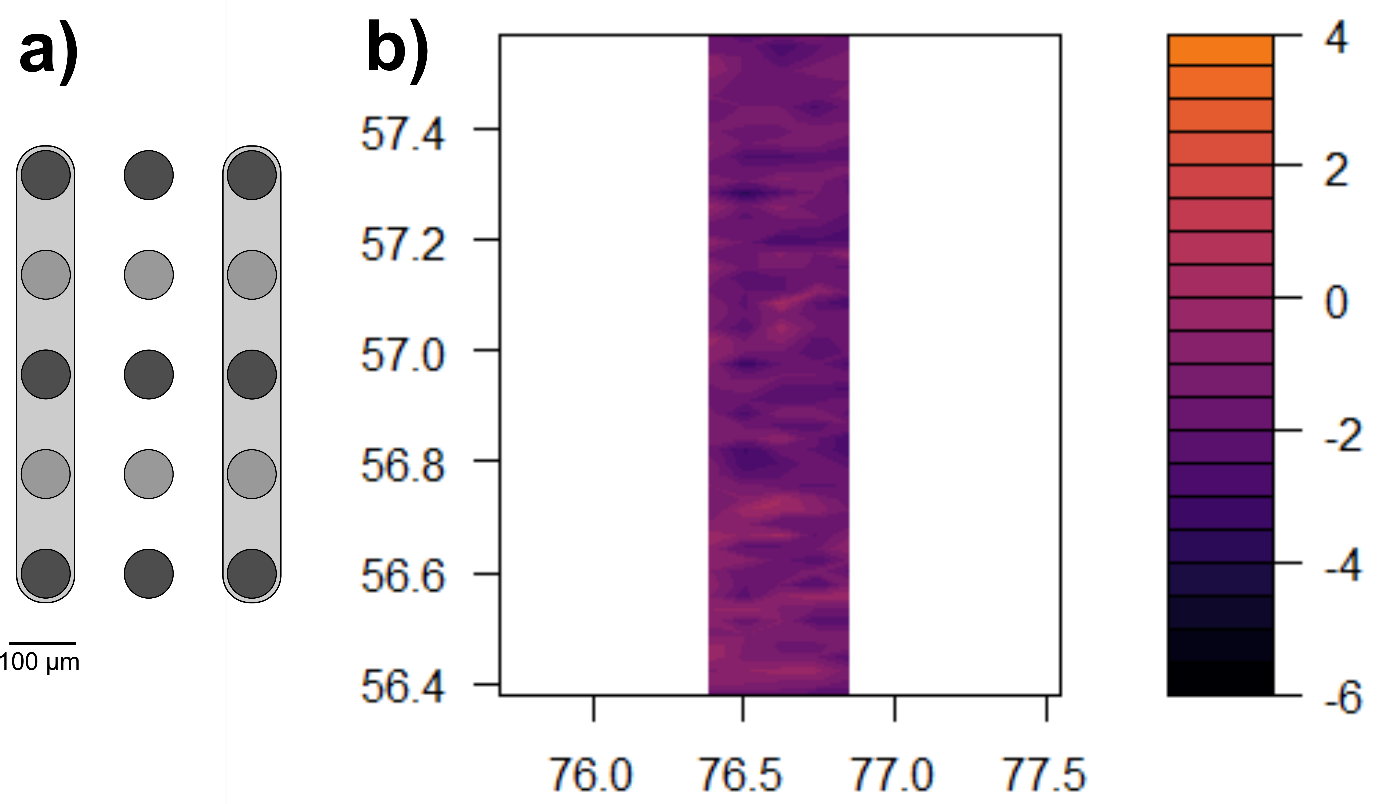


**Figure S4.** NIST Porosity test, a) Schematic map of NIST porosity ‘pit-test’, pore like wells were pre-ablated into a slab of NIST SRM612 glass. Light grey trenches (~20 µm deep), mid-grey pits (~150 µm deep), and dark grey pits (~300 µm deep) mimic the structures within the coral. b) resulting δ^11^B map of ablated area, performed analytically and statistically in the same way the coral maps. The shown scale is in mm and the variation in ‰ in the colour scale.

**
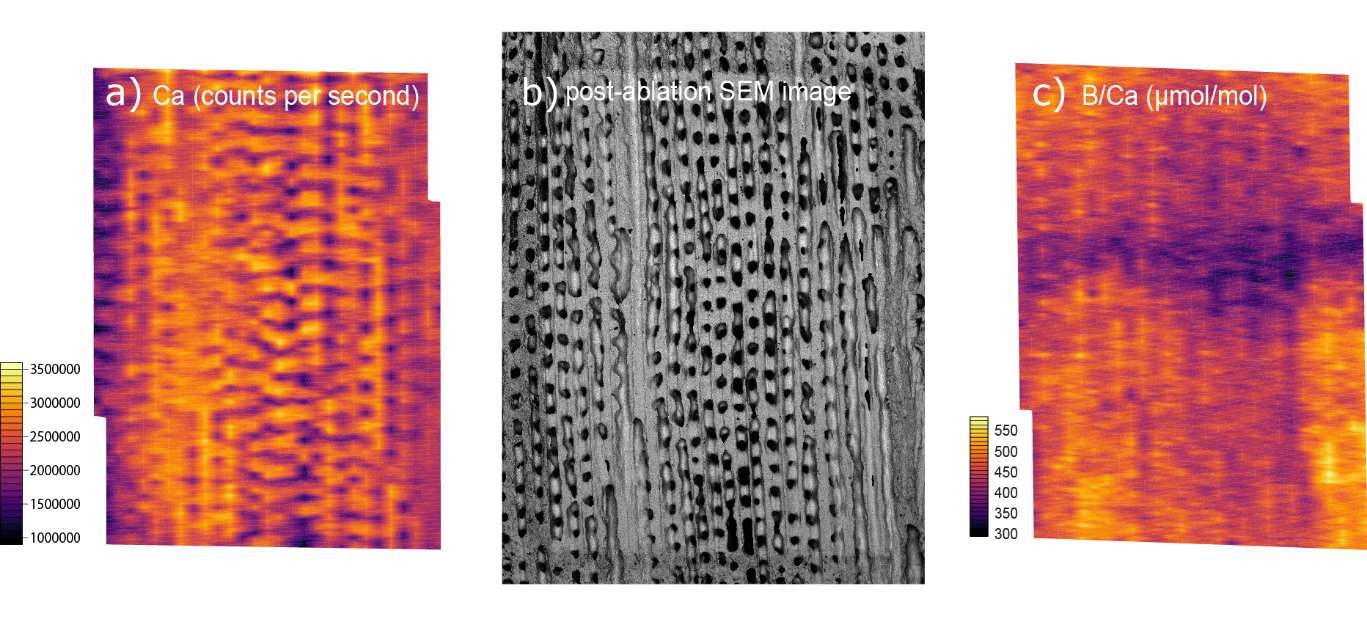
**

**Figure S5.** Comparison of LA signal intensity against geochemical data and sample geometry, a) Ca counts (per second) from across the mapped region. This map shows the density and structure of the coral well, as the pores result in a decrease in Ca counts. b) Post-ablation SEM image shows the structure and increased porosity of the coral in the columella sections. c) B/Ca (μmol/mol) of the measured section, there is no systematic relationship between this and the Ca counts in a) showing that the signal intensity and visible porosity are not impacting the element ratios produced.

**
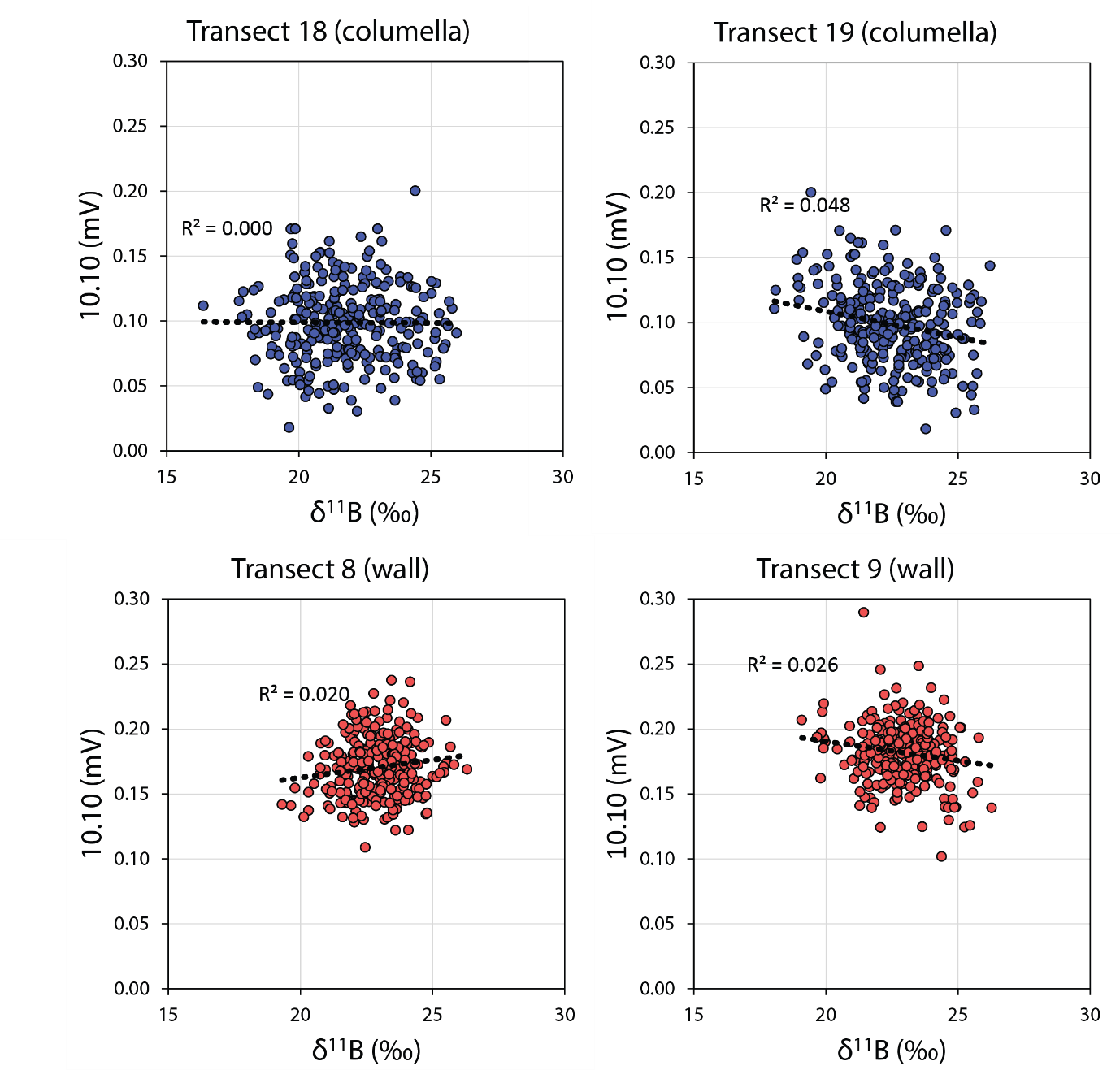
**

**Figure S6.** Plots comparing Ca_interference_ (which can be viewed as a proxy for the amount of sample material being ablated) intensity (~10.10 amu) against δ^11^B (‰) for four different transects across the analysed coral, two columella sections and two wall sections. The difference in the amount of carbonate ablated is clearly visible in the 10.10 intensity between the two components, which captures the difference in porosity between the two structural elements. None of the transects show any relationship with the δ^11^B measured, which is suggestive that porosity does not impact the measurement of the carbonate skeleton through either topographic or changing matrix effects.
